# Circadian regulation of innate immunity in animals and humans and implications for human disease

**DOI:** 10.1007/s00281-022-00921-z

**Published:** 2022-02-15

**Authors:** Joanna Poole, Gareth B. Kitchen

**Affiliations:** 1 Southmead Hospital, North Bristol Trust, Southmead Rd, Bristol, BS10 5NB UK; 2 Faculty of Biology, Medicine and Health, University of Manchester, Manchester Academic Health Sciences Centre, Manchester, M13 9PT UK; 3 Manchester University NHS Foundation Trust, Manchester Academic Health Science Centre, Manchester, M13 9WL UK

## Abstract

Circadian rhythms are 24-h oscillating variations in physiology generated by the core circadian clock. There is now a wide body of evidence showing circadian regulation of the immune system. Innate immune cells contain the molecular circadian clock which drives rhythmic responses, from the magnitude of the inflammatory response to the numbers of circulating immune cells varying throughout the day. This leads to rhythmic presentation of disease clinically, for example the classic presentation of nocturnal asthma or the sudden development of pulmonary oedema from acute myocardial infarction first thing in the morning.

## Introduction

The earth’s rotation provides cyclical light:dark phases that have provided contrasting environments for cellular life to partition its pathways, especially those of metabolism and immune defence. Whilst it is posed by some that circadian rhythms evolved from peroxiredoxin responses [28] to oxidative solar stress, they are a feature in all three domains of life — eukaryotic, prokaryotic and archaea [28]. These rhythms can be intrinsic (cycling patterns of protein synthesis and degradation), light-driven or intrinsic with modulation by feeding. Examples can be found across the animal kingdom.

The innate immune system is an early line of defence against pathogen exposure and does not require previous exposure or ‘memory’ of the pathogen to be effective. Features of it persist across the animal kingdom [75]. It consists of antimicrobial peptides [50], pattern recognition receptors [41], cytokines [98], complement [25] and phagocytic cells [35]. In higher animals, the presence of cells with oxidative killing (e.g. neutrophils) can lead to tissue damage [51, 67]. Given this oxidative stress, and the origin of the clock as an oxidative stress buffer, circadian modulation of innate immunity has been proposed and studied, in animals.

A landmark paper by Halberg in 1960 [39] demonstrated that lethality in a mouse model of inhaled endotoxin (lipopolysaccharide) varied depending on time of day. The Ray group has shown that knocking out the clock component *Bmal1* in mouse myeloid cells [48] is protective against streptococcal pneumonia. Other studies have gone on to confirm that caecal ligation puncture in mice also has a circadian susceptibility — worse in the dark phase [40]. Similar has been shown in the flounder fish [114] — *Bmal1* knockout enhances pro-inflammatory cytokines and improves survival in bacterial infection [114]. Moreover, illumination at night has been shown to affect both clock genes and inflammatory cytokines in zebra finches [4, 70].

The therapeutic interest in clock targets is their modulation of the inflammatory response, reviewed in this article, and mediation of oxidative stress defences. Because of the therapeutic value of these targets to human drug development, the review will focus on mammalian circadian circuits and their impact on the innate immune system.

In particular, photic regulation via the suprachiasmatic nucleus (SCN) and cell clock gene regulation of myeloid behaviour will be detailed, because these provide translational targets (Reverbα and RORα have agonists available); information about the sympathetic nervous system, circadian rhythms and inflammation, is a large topic and was reviewed by Leach and Suzuki [56] recently. Lastly, entrainment of the liver clock via feeding cues will NOT be provided, because this is an ongoing topic and has been provided a mini-review very recently here [72].

## Feedback regulation

**Fig. 1:**
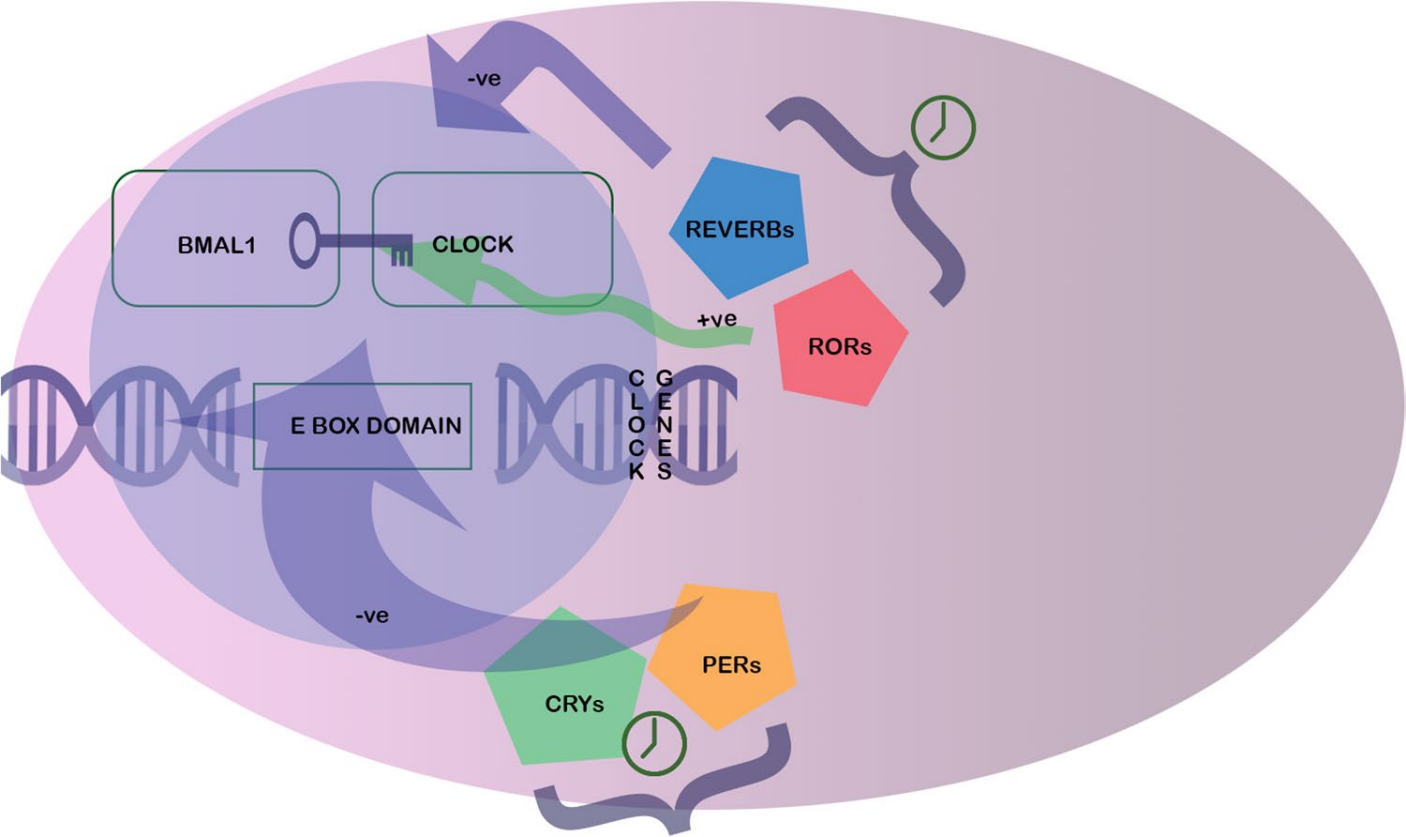
Schematic of BMAL1:CLOCK gene transcription and feedback

The molecular circadian clock consists of two interlocking transcription-translation feedback loops (TTFL) that converge on BMAL1 and CLOCK [93]. The basic mechanism involves transcriptional activation and repression, allowing rhythmic activation and repression of target genes. The transcriptional activators are BMAL1 and CLOCK, and the repressors are PERIOD (PER1, PER2 and PER3) and CRYPTOCHROME (CRY1 and CRY2). The BMAL1/CLOCK heterodimers also promote transcription of the nuclear receptors REVERBα/β and RORα [17] which form accessory repressive and activating loops respectively (Fig. 1).

## Suprachiasmatic nucleus

The suprachiasmatic nucleus in mammals is considered the pacemaker circuit; lesions produce changes in sleep [113], circadian rhythm [27] and melatonin output [89]. Ex vivo SCN neurons still show phasic output [105]. A considerable proportion of SCN neurons are light responsive [38] — their electrical activity coincides with the light phase [68]. Light exposure entrains SCN output [23].

The SCN network’s output has a sinusoidal pattern; however, its relationship with behaviour depends on whether the animal is nocturnal (SCN activity nadir occurs with active phase) or diurnal (SCN zenith occurs with active phase) [16].

A number of clock genes within the SCN are light responsive, for example *Per1* and *Per2* [95]. This has raised concerns within ecology for the effect of dim evening light on urban animals — for example birds. Dim light at night has been shown to alter *per2* expression in the hypothalamus of zebra finches [4], in addition to reducing *tlr4* and *il-1β* mRNA transcripts. In association was loss of standard 24-h cycles of *clock*, *ror-α* and *cry1*. The emphasis of dim light at night in zebra finches was confirmed to alter cytokines *IL-1β* and *IL-10* in a separate study [70].

The effect of clock-gene changes on cytokine expression is not unique to birds and has also been demonstrated in zebra fish, where the genes *period1* and *period2* alter cytokine expression and *per1b* alters neutrophil recruitment [88].

There is consistency in mammals regarding photic influence of immunity. For example, mice kept in constant dark conditions show three times the mortality of those with a typical light:dark cycle, although this is independent of the myeloid expression of CLOCK or BMAL-1 [54]. Furthermore, mice in a caecal ligation puncture (CLP) model of sepsis demonstrate less severe sepsis and organ injury when exposed to high-illuminance blue light [59], which mimics early morning light. The same paper discusses a small number of human patients with appendicitis exposed post-operatively to blue light and found a significant reduction in cytokines such as IL-6 and IL-10, although the number was too small to comment on clinical outcomes.

One of the key differences in mammals is whether they are diurnal (like humans) or nocturnal, e.g. mice and hamsters. This is important because it has previously been shown that BMAL1 and PER2 in humans/mice are antiphase from one another in expression [58]. This may reflect that circadian regulation reflects behaviour/activity in an organism rather than simply external night or day — in the case of immunity, pathogen exposure and injury are of course more likely when out hunting/foraging/socialising, than sleeping.

In the case of rats, both constant dark and constant light produced worse mortality in caecal ligation puncture sepsis than a standard light/dark cycle [13]. Standard 1-week mortality was 83% in cycling conditions, 62.5% constant light and 31% constant dark. In these rats, non-survival was associated with an early peak cortisol in relation to plasma ACTH — the authors’ conclusion was external light cues modified the hypothalamic–pituitary–adrenal axis. Consistent with this is similar evidence in mice that constant dark conditions exacerbate sepsis lethality [54], and for these authors, this change was independent of myeloid clock genes, suggesting a clock gene-exogenous pathway.

Interestingly, human shiftworkers have been shown to have an increased susceptibility to infection [63, 64] which may in part be explained by the light-at-night phenomenon seen in other species (mammals, birds and fish). There are also important implications for patients, who often experience dim lighting rather than true dark conditions, especially in intensive care where monitors and procedures interrupt ‘dark’ conditions.

That said, interventions attempting to re-entrain patient circadian rhythms with light have only been done in a small number of patients in critical care, measuring a melatonin metabolite; the study contained 22 patients and suffered with attrition, although it did show an improved phase/synchronisation [34], but was unable to comment on clinical outcomes. Other interventions using light exposure have not been shown to affect a particular outcome of concern, delirium [96, 97].

We will go on to discuss circadian behaviour in innate cell subsets, with respect to neutrophils and macrophages. Further reviews with a wider scope have been published [5, 12].

## Neutrophils

Neutrophils are phagocytic cells, whose origins may have been as far back as cnidarians — evidence of phagocytotic activity is evident in some species of coral [78]. In humans, up to 10^10^ neutrophils are produced daily [100]. Number and percentage of neutrophils appear to vary by species — in humans, they represent 40–50% of the total leukocyte pool, whilst this can be considerably different in other species [33]. There is little published data on circadian immunology in cnidarians, who do however have light responsive transcriptome responses [55]. However, there is evidence in mice [1], pigs [31] and humans [30] that neutrophil behaviour has a daily rhythm.

Features of neutrophil behaviour displaying circadian rhythms include egress under CXCR4 in humans [30], the NADPH oxidase enzyme used in oxidative burst killing [30] and phagocytosis [43] — reduced by 40% in constant light conditions in mice [43].

**Fig. 2:**
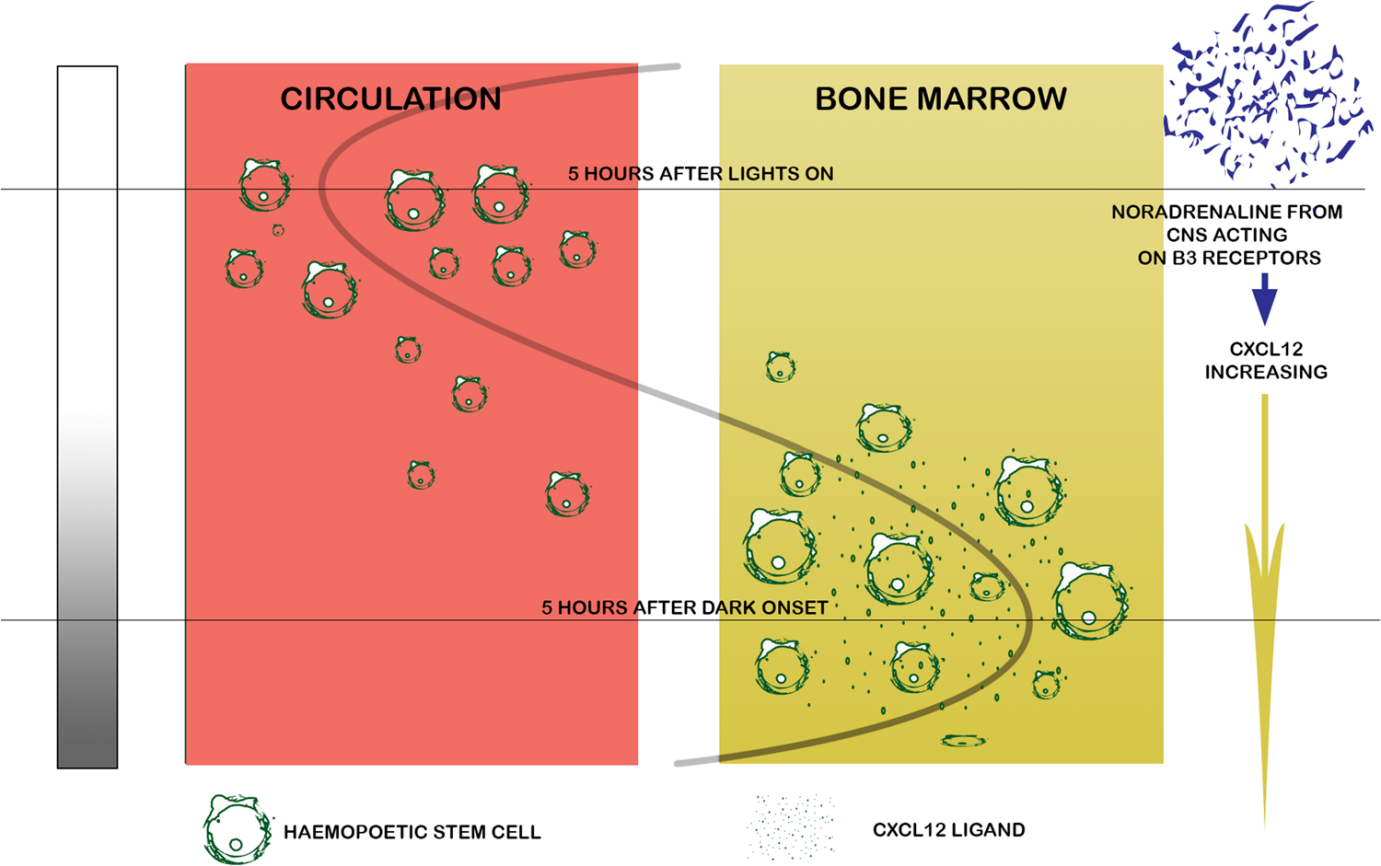
Diagram from Méndez-Ferrer et al. [69] demonstrating circadian regulation of circulating haematopoetic cells by CXCL12, via noradrenaline

In mice, neural output through the sympathetic nervous system via noradrenaline affects expression of CXCL12 via B3 mediated SF-1 expression (Fig. 2) [69]. CXCL12 is a homing signal for the neutrophil receptor CXCR4 as well as important for bone marrow haematopoetic stem cells [99], and further reviewed recently as a key determinant of neutrophil trafficking [22]. Moreover, CXCR4 antagonists mobilise the haematopoetic stem cell pool in both mice and primates [32].

Evolutionarily, CXCR12 and CXCR4 are considered antecedent to a sophisticated immune system, with extant chemokines potentially deriving from the CNS [45]. CXCR4 and CXCL12 both have a role in the development of the nervous system [71], and *Cxcr4* knockout mice, in addition to high embryonic lethality, display profound defects in marrow haemopoesis and nervous system development [65]. Human CXCR4/CXCL12 signalling shares conservation with mice, and human *CXCR4* knockin restores some of the leukocyte, and neutrophil, features of knockout mice [19]. This mouse line has been proposed as useful for CXCR-4-based human therapies. Noradrenaline has a circadian rhythm, as does adrenaline [61], and their complex interactions beyond the scope of this review have been reviewed excellently by Leach and Suzuki [56]. The effect of such catecholamines on myeloid cells may explain why adrenalectomy removes diurnal rhythms in circulating blood leukocytes in mice [9].

One of the pathogen defences of neutrophils is NETosis — the production of neutrophil extracellular traps (NETs) which consist of chromatin and antimicrobial molecules [8]. Mature neutrophils have a circadian ‘responsiveness’ in NETosis, driven by CXCL2, in mice [2]. The circadian responsiveness was replicated in humans and correlated with pneumonia severity — offering a potential therapeutic or chronotherapeutic pathway [2].

The avian analogue of neutrophils, heterophils, is also observed to have a diurnal acrophase [66], whilst neutrophil injury recruitment in fish also has a circadian phenotype [87] suggesting these features are well preserved in different animals. The latter is influenced by melatonin, which has extensive pathways, reviewed with respect to immunity and inflammation previously [12, 14, 101, 110]. Examples of how melatonin may influence immunity, however, through increasing the weight of immune organs [81], reducing neutrophil apoptosis [73] and increasing neutrophil burst killing [82].

## Macrophages

Macrophages are phagocytes of the innate immune system with tissue-specific fates and phenotypes [111]. They circulate as monocytes for a number of days, before maturing into macrophages on receipt of external cues, when they may develop different subtypes [111]. In addition to recruitment to sites of infection and damage, they are an intrinsic component of wound and tissue healing and regeneration [111]. They also have circadian transcriptomes and behaviours which affect function [46]. Post-translational circadian regulation of macrophage function is also seen, especially with respect to metabolic networks and mitochondrial morphology [18].

Keller et al. established that spleen and lymph node-derived macrophages contain autonomous cellular oscillators with 8% of their transcriptome being expressed in a circadian pattern [46]. Essential elements of importance are the lipopolysaccharide (LPS) receptor TLR4, TNFα pathway and other LPS-associated receptors such as CD14, MAPK14 and AP-1 subunits JUN and FOS, as well as ADAM 17.

Analysis of clock genes by Keller et al. showed that PER2 and REVERBα display high-amplitude oscillations with 4- and 20-fold differences, respectively, at peak and trough levels in macrophages. In peritoneal macrophages especially, mRNA transcripts of PER2 and REVERB varied by as much as 100–300-fold. The corresponding changes in cytokine production (IL-6 and TNFα) showed threefold changes. These changes persisted even with removal or addition of glucocorticoid mediators [46].

In addition to variation in mRNA transcripts, absolute splenocyte counts vary in a circadian fashion in Keller’s study, demonstrating regulation of leukocyte trafficking.

Kitchen et al. demonstrated that clock knockout of the gene *Bmal-1* in mice in macrophages presented a survival advantage in streptococcal pneumonia [48], partly through recruitment and phagocytosis. Thus, a basal fluctuation in circadian behaviour may tend towards important survival advantages.

Intriguingly, more recent work in mice has demonstrated that the polarisation state of the macrophage (M1 or M2) is associated with differential periodicity and amplitude in expression of clock genes themselves [115]. In the M1 state, transcripts of BMAL1 and PER2 are suppressed, with normal periodicity (just the amplitude changes), whilst in the M2 state, periodicity is lengthened, whilst amplitude remains the same. This suggests an association between clock gene expression and behaviour — a pathway that may be target for treatments of inflammatory human disease, or even wound healing. For example, wound healing in Siberian hamsters has a clear circadian rhythm [10], whilst shifts in photoperiod reduce the number of M2-polarised macrophages in adipose tissue in mice [47].

This has direct implications for human shiftworkers, who have higher rates of obesity, diabetes, cardiovascular disease and cancer [90]. Other more novel implications are those regarding shiftwork, reduced fertility [108] and miscarriage [6]. As pro-inflammatory macrophages have been shown to affect number and quality of ovarian follicles [62] and successful embryonic development [112], there is urgent need to clarify therapeutic targets in this group of workers. In 2018, in data published by TUC (Trade Union Congress) in the UK alone, night workers counted for more than 3 million employees, or 1 in 9 workers, with the female proportion increasing by more than 100,000 in the preceding 5 years [102]; thus, this represents a considerable health burden.

## Targeting clock genes in sepsis and other inflammatory diseases

This section will focus on REVERBα and RORa agonists as these are available and have some pre-clinical use.

Sepsis has been well studied in mouse models through a circadian lens. This began with the very early experiments by Halberg et al. [39] noting a time-of-day lethality to inhaled endotoxin.

More recently, this has been observed in caecal ligation puncture models of sepsis in murine models [40]. Mechanistically, this appears to coincide with cytokine levels, e.g. IL-6 — perhaps because the polymicrobial contamination in the peritoneum provoked the ‘cytokine storm’ that produces a SIRS response and multi-organ failure. Further experimentation revealed that a mutation in *Per2* in these mice removed the circadian lethality, demonstrating a pathway under PER2 regulation mediates this effect.

Aside from direct cytokine effects, further circadian influence over sepsis severity could potentially relate to activity of the inflammasome — an IL-1-producing assembly. The receptor portion of this assembly of proteins is encoded by *NLRP*. REVERBα, the circadian nuclear receptor, regulates production of this mRNA [84]. The inflammasome has recently been reviewed in sepsis [21, 109], as well as the epidemic virus SARS-CoV-2 [80, 103]. This poses the idea that circadian regulators may provide important basal resistors/enhancers of the inflammasome and therefore many inflammation-based diseases.

Another older study in hamsters demonstrated that photoperiod affected lethality to endotoxin [85] — short days were protective in comparison to long days. Animals exposed to short days had lower cytokine levels than their comparators. As with other studies on endotoxin [11], females survived long-day exposure better than male counterparts.

Together, these experiments, and those mentioned earlier in the review, demonstrate the influence of light and clock genes on components of the immune system and why they may be excellent targets for therapy.

The clock gene *RORa* has been shown to be a negative regulator of inflammatory behaviour in human macrophages [74]. When *RORa* is deleted, IL6, TNFa and IL-1 expression increase — the authors of this study propose therefore that *RORa* regulates the basal inflammatory state of macrophages. This idea is subserved by its role in murine models of inflammatory bowel disease, where deletion predisposes to chronic inflammation [77].

Moreover, a role in negatively regulating neutrophil activity and recruitment has been identified for *rora* in zebrafish [44], suggesting conserved relationships between clock genes and immune function.

Even more convincingly, a genome-wide association study in 28 human intensive care patients with sepsis identified blood leukocyte *RORa* as under-expressed and delayed in restoration, in high-severity versus low-scoring sepsis patients [15]. This is further corroborated by a gene and network analysis study performed in public data of paediatric sepsis patients [79], identifying RORa as one of fifteen master regulators that influence sepsis severity. The authors of this study highlight that downregulation of these master regulators is causal for the sustained state of inflammation seen in severe sepsis.

REVERBα has also gained interest as a target in inflammatory diseases. Knockout models have shown us that loss of *Reverbα*, or its mutation, is pro-inflammatory. A knockout model demonstrates increased neuroinflammation and microglial activity (an innate immune cell of the CNS) that could be attenuated with the REVERBα agonist SR9009 [37]. Meanwhile, a mouse model of pulmonary inflammation showed that mutating *Reverbα* causes increased pulmonary responsiveness and aggression in myeloid cells [17], whilst mutating its paralogue *Reverbβ* in bronchoepithelial cells also enhanced inflammation. Moreover, the inflammation itself caused changes in stability and degradation of REVERBα via SUMOylation and ubiquitination, showing there is a reciprocal relationship between inflammation and circadian rhythm, which could result in a wind-up phenomenon. Similarly, Durrington et al. confirmed REVERBα as a gateway to asthma response, reflecting the importance of this pathway for pulmonary disease [26].

Gibbs et al. confirm that reduction in REVERBα increased IL-6 and other cytokines in human and mouse macrophages, whilst another group demonstrated that SR9009 (Reverbα agonist) improves survival in a murine caecal ligation puncture model of sepsis [36], suggesting through mixed REVERBα and light studies that this was a pathway through which blue light improves survival in their model of *Klebsiella pneumonia* in mice.

Other roles for SR9009 have been found in cardiovascular ischaemia [86], inflammasome inhibition [42] and reducing the LPS-driven M1 polarisation of macrophages in pregnancy loss [20], demonstrating a persistent role for REVERBα in inflammation.

## Sexual dimorphism

Exemplified recently by the higher male lethality of COVID-19 [7], it has been recognised for some time that there is a sex difference in infection survival [3, 104] in addition to a sex difference in autoimmune disease, where 80% of patients are female [91]. Since there are also circadian differences in the two biological sexes, for sleep [106] and cycle length [24], there is growing interest in how sex and circadian rhythm intersect to affect immunity. There is not much published information as this is a relatively novel field.

There are sex hormone receptors in the human suprachiasmatic nucleus itself [52] and these are thought to help modify electrical activity in response to photoperiod/jet lag (female mice show faster photic entrainment [53]; the evolutionary benefit in a sex difference may be the female’s increased adaptability for childcare needs.

As has already been reviewed in other sections, there is evidence across species that the SCN/light input influences cytokine titres, so it is feasible that sex hormones influence immunity, via sex steroid receptors in the SCN itself.

A pre-eminent mechanism, however, might be the evidence that sex hormones differentially regulate RORa (reviewed in a previous section). Oestrogen increases its activity whilst testosterone reduces it [92]. This could explain the preponderance of Th17-mediated diseases in female (humans) like multiple sclerosis [29], as well as a worse outcome from endotoxin exposure in male mice, and sepsis in human patients, although these all are likely to be multifactorial.

Sex hormones are likely involved as confirmed by circadian and sleep changes in both menstrual phase in mammals [94] and pregnancy [76, 107].

There is also evidence of sexual dimorphism in humans of PER2 expression in the central nervous system [60], as well as altered amplitude in rhythmic expression in the murine adrenal gland [49] — evidence for adrenaline/noradrenaline in influencing immune behaviour has been mentioned with respect to bone marrow pools, and reviewed in detail here [56].

Thus, there is a range of mechanisms by which sexual dimorphism in circadian immunity could be made manifest. This field is evolving.

## Discussion

It is clear that the regulation of inflammation is finely balanced in health, with choreographed gene expression and tissue behaviour, modified by clock genes. Many of these pathways interact with each other, either through light, or the sympathetic nervous system, and can be rewired in inflammation. They are often conserved across animal kingdoms, suggesting they are important.

It appears that there are many ways the circadian immune system is influenced — be it gene expression secondary to native clock gene oscillation, output from the SCN via melatonin, or adrenaline and noradrenaline, as well as corticosteroids, which were not reviewed here.

The field has developed from observation of circadian immune phenomena — like severity of response to pathogen exposure — to clarification of the cytokines involved, the recruitment and killing capacity of innate cells (e.g. burst killing, phagocytosis and NETosis), and the development of gene-manipulated mice. Evidence linking light, or circadian gene expression, to cytokines and disease survival has been provided in mammals, birds and fish. New gene sequencing and chromatin structure sequencing has allowed researchers to describe mechanistic links between light or time of day and protein expression. Proteome analysis has shown that inflammation can have reciprocal effects on protein stability and how inflammatory disease may rewire circadian circuits [83].

Furthermore, new translational drug opportunities have appeared with targets for REVERBα and RORα, which show promise in pre-clinical models.

Limitations in our knowledge are the huge complexity of multiplexed regulation — the intersection of light, baseline gene fluctuation, feeding status, sterile or non-sterile inflammation, and the sympathetic nervous system. Furthermore, we also rely on ex vivo analysis of human cells, which no longer have access to the full complement of regulators available in vivo.

Moreover, many of our pre-clinical studies are based on mice, who are typically nocturnal. This may mean that alterations in their immune systems following ‘resting phase’ interventions by human lab workers could contribute to the well-known failure of pre-clinical studies to translate into successful human therapies [57].

Dysregulated innate immunity is the basis for a number of inflammatory conditions that are known to affect humans. The effect of light and shiftwork, therefore, on innate immunity has large health implications for shiftworkers, as well as reproductive health in women.

Furthermore, the effect of light on circadian dysrhythmia and immune systems has implications for ecology, e.g. light in urban areas, as well as for humans exposed to artificial lighting, both in health and disease.

Future directions may focus on translating pre-clinical therapies into humans, understanding the complex interplay of different in vivo systems — which may require computer network algorithm analysis — and the effect of sexual dimorphism, and age, on all these pathways.

Given such a diverse array of diseases have demonstrable circadian rheostats, it is essential that we deepen our understanding of such pathways, to target treatments, and reduce medical and ecological burdens in a world where technology is competing with life’s reliance on environmental rhythms.
